# Supplementary material for: 90-Day Patient-Centered Outcomes after Totally Endoscopic Cardiac Surgery: A Prospective Cohort Study
Source: J Clin Med. 2022 May 9;11(9):2674. doi: 10.3390/jcm11092674 (PMC9103144; doi:10.3390/jcm11092674)
Supplement: Supplementary file 1 [file jcm-11-02674-s001.zip › jcm-1691803-supplementary.pdf]

Supplementary

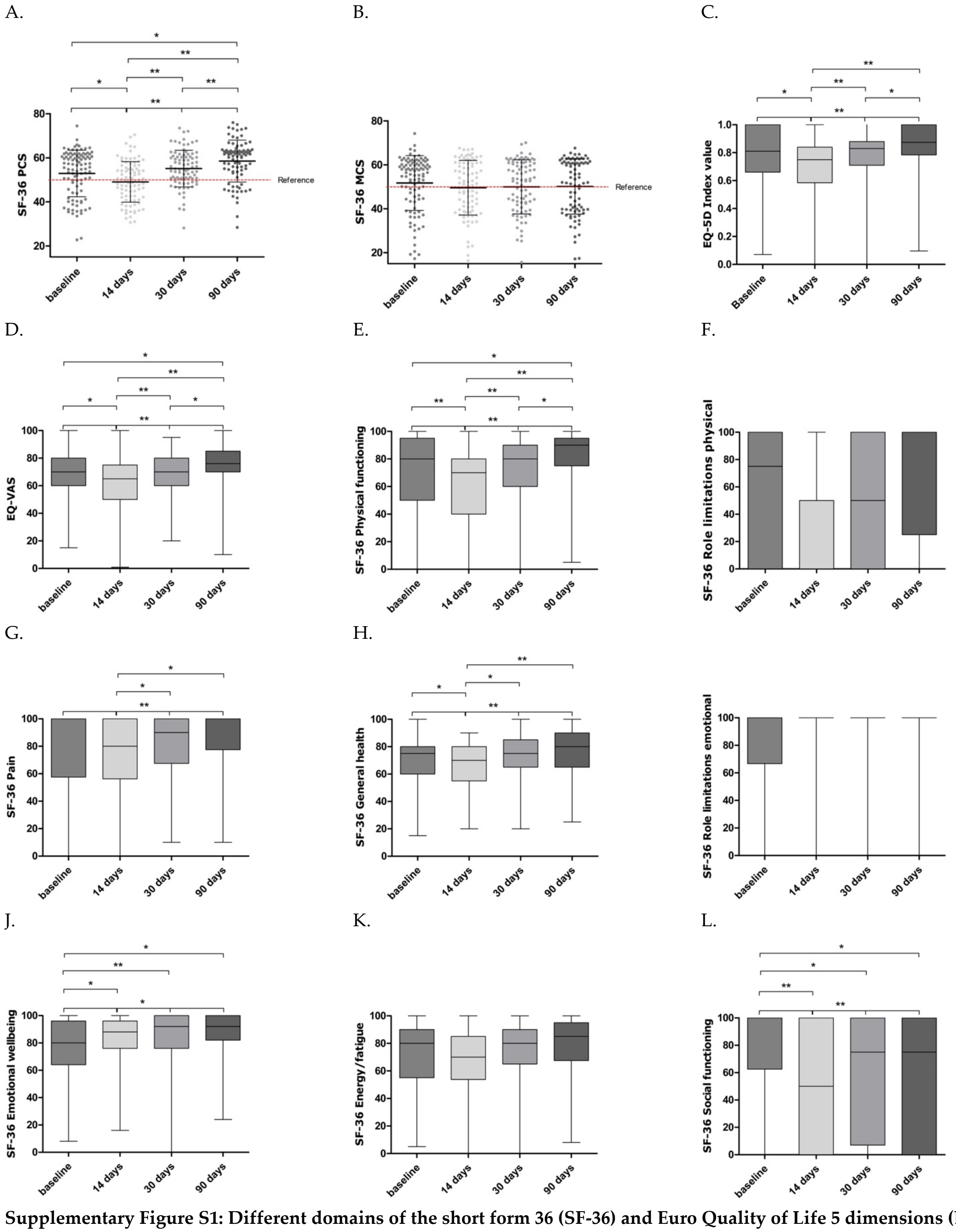

Supplementary Figure S1: Different domains of the short form 36 (SF-36) and Euro Quality of Life 5 dimensions (EQ-5D) questionnaires after endoscopic coronary artery bypass grafting. Data are shown as median and interquartile ranges. Significance is tested with the Friedman and Wilcoxon Signed Rank test with Bonferroni correction and is indicated as *: $p < 0.05$; **$p < 0.001$. MCS: mental component score, PCS: physical component score, VAS: visual analogue score.

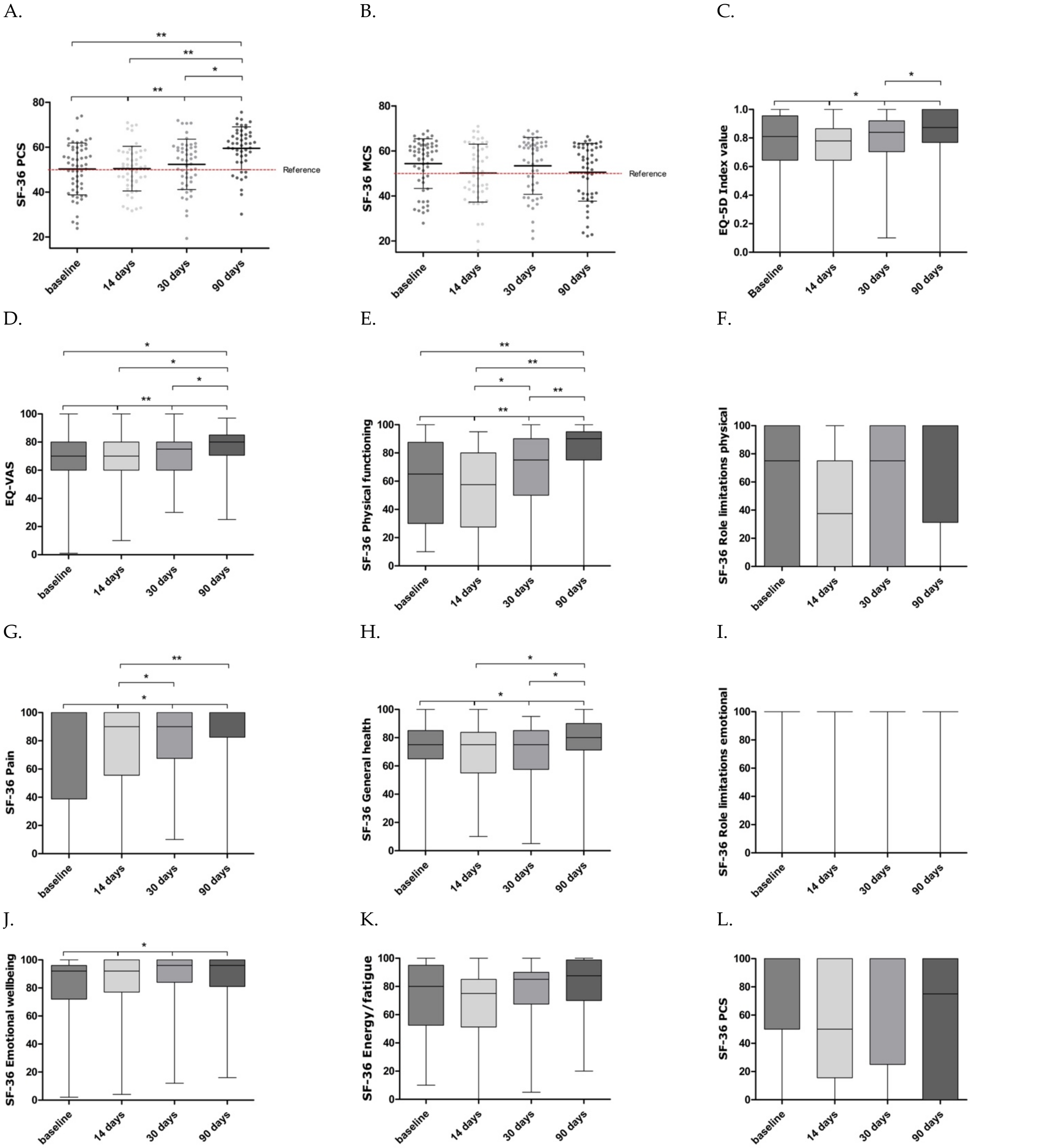

Supplementary Figure S2: Different domains of the short form 36 (SF-36) and Euro Quality of Life 5 dimensions (EQ-5D) questionnaires after endoscopic aortic valve replacement. Data are shown as median and interquartile ranges. Significance is tested with the Friedman and Wilcoxon Signed Rank test with Bonferroni correction and is indicated as *: $p < 0.05$; **$p < 0.001$. MCS: mental component score, PCS: physical component score, VAS: visual analogue score.

Supplementary Table S1: Recovery after 30-days and improvement after 90-days.

	TECS (n=193)	Endo-CABG (n=99)	Open CABG (n=8)	Yil-AVR (n=57)	TAVI (n=8)	MVATS (n=16)	Combination (n=23)
**30-days**							
Not recovered	87 (45.01)	38 (38.38)	6 (75.00)	23 (40.35)	3 (37.50)	5 (31.25)	13 (56.52)
Recovered	100 (51.81)	50 (50.50)	2 (25.00)	28 (49.12)	5 (62.50)	9 (56.25)	7 (30.43)
Improved	64 (33.16)	31 (31.31)	1 (12.50)	18 (31.58)	5 (62.50)	6 (37.50)	4 (17.39)
**90-days**							
Not recovered	54 (27.98)	30 (30.30)	4 (50.00)	13 (22.81)	2 (25.00)	5 (31.25)	2 (8.70)
Recovered	124 (64.25)	57 (57.57)	4 (50.00)	37 (64.91)	6 (75.00)	9 (56.25)	13 (56.52)
Improved	88 (45.60)	42 (42.42)	2 (25.00)	24 (44.11)	3 (37.50)	7 (43.75)	11 (47.83)

CABG: coronary artery bypass grafting; Endo-CABG: endoscopic coronary artery bypass grafting; MVATS: mitral valve repair or replacement through video-assisted thoracoscopic surgery; TAVI: transcatheter aortic valve implantation.; TECS: totally endoscopic cardiac surgery; Yil-AVR: endoscopic aortic valve replacement.

Supplementary Table S2: Univariate factors tested for correlation with 30-day recovery and 90 day improvement.

	**Coefficient**	**SE**	**OR**	**95% CI**	**P-value**
A. Univariate factors tested with 30-day recovery					
**Age**	-0.040	0.018	0.960	0.928 – 0.994	**0.021**
**CPB time**	-0.009	0.004	0.991	0.983 – 0.999	**0.022**
**AHT**	-0.711	0.341	0.491	0.252 – 0.958	**0.037**
**Smoking**	-	-	-	-	**0.048**
**Euroscore II**	0.167	0.085	0.849	0.719 – 1.002	**0.053**
**ICU LOS**	-0.006	0.003	0.994	0.988 – 1.000	**0.052**
**Hospital LOS**	-0.071	0.038	0.931	0.864 – 1.004	**0.062**
**Education**	-	-	-	-	**0.075**
**Combinations**	-0.879	0.497	0.415	0.157 – 1.101	**0.077**
**Clamping time**	-0.008	0.005	0.992	0.982 – 1.001	**0.091**
Transfusion packed cells	-0.681	0.511	0.506	0.186 – 1.377	0.182
Revision <48h	-0.700	0.747	0.497	0.115 – 2.149	0.349
Gender	0.327	0.368	0.721	0.350 – 1.48	0.375
EQ-5D Pain at 14 days	-	-	-	-	0.534
Bleeding 24h	0.000	0.000	1.274	0.999 – 1.000	0.590
Diabetes mellitus	-	-	-	-	0.801
BMI	0.007	0.037	0.993	0.923 – 1.068	0.849
Revision < 1 week	21.412	20096.49	0.000	0.000 – 0.000	0.999
Neurological complications	-	-	-	-	1.000
B. Univariate factors tested with 90-day improvement					
**BMI**	0.073	1.064	0.127	0.997 – 1.159	**0.059**
**Hospital LOS**	-0.076	0.042	0.927	0.854 – 1.006	**0.070**
**Clamping time**	0.008	0.005	1.008	0.999 – 1.018	**0.094**
Gender	-0.550	0.364	0.577	0.283 – 1.177	0.130
Euroscore II	-0.101	0.81	0.904	0.771 – 1.060	0.214
ICU LOS	-0.002	0.002	0.998	0.994 – 1.001	0.217
AHT	0.383	0.332	1.467	0.766 – 2.812	0.248
Bleeding 24h	0.000	0.000	1.000	1.000 – 1.001	0.325
EQ-5D Pain at 14 days	-	-	-	-	0.441
CPB time	0.003	0.004	1.003	0.995 – 1.010	0.482
Transfusion packed cells	0.330	0.502	1.391	0.520 – 3.718	0.511
Combinations	0.309	0.479	1.362	0.533 – 3.480	0.519
Diabetes mellitus	-	-	-	-	0.525
Revision < 1 week	1.050	1.165	0.350	0.036 – 3.435	0.755
Education	-	-	-	-	0.808
Smoking	-	-	-	-	0.818
Age	0.003	0.017	1.003	0.971 – 1.036	0.848
Revision <48h	0.075	0.725	1.078	0.261 – 4.460	0.918
Neurological complications	-	-	-	-	1.000

AHT: arterial hypertension; BMI: body mass index; CPB: cardiopulmonary bypass; Euroscore II: European System for Cardiac Operative Risk Evaluation; EQ-5D: Euro Quality of Life 5 dimensions; ICU: intensive care unit; LOS: length of stay
